# New Technologies With Increased Precision Improve Understanding of Endothelial Cell Heterogeneity in Cardiovascular Health and Disease

**DOI:** 10.3389/fcell.2021.679995

**Published:** 2021-08-27

**Authors:** Ashley Dawson, Yidan Wang, Yanming Li, Scott A. LeMaire, Ying H. Shen

**Affiliations:** ^1^Division of Cardiothoracic Surgery, Michael E. DeBakey Department of Surgery, Baylor College of Medicine, Houston, TX, United States; ^2^Department of Cardiovascular Surgery, Texas Heart Institute, Houston, TX, United States

**Keywords:** endothelial cell heterogeneity, transcriptomics, endothelial mesenchymal transition, vascular remodeling and arteriogenesis, vascular disease, atherosclerosis, cardiac disease

## Abstract

Endothelial cells (ECs) are vital for blood vessel integrity and have roles in maintaining normal vascular function, healing after injury, and vascular dysfunction. Extensive phenotypic heterogeneity has been observed among ECs of different types of blood vessels in the normal and diseased vascular wall. Although ECs with different phenotypes can share common functions, each has unique features that may dictate a fine-tuned role in vascular health and disease. Recent studies performed with single-cell technology have generated powerful information that has significantly improved our understanding of EC biology. Here, we summarize a variety of EC types, states, and phenotypes recently identified by using new, increasingly precise techniques in transcriptome analysis.

## Introduction

Endothelial cells (ECs) are vital for maintaining blood vessel integrity in the cardiovascular system. Beyond functioning as a physical barrier, ECs have been shown to play a broader role in vascular physiology and pathology, exhibiting various phenotypes associated with different functions. In addition, ECs have been shown to change phenotype in response to tissue injury, shear stress, physical location, and environmental stimuli. Changes in ECs at the transcriptional level have been compared between healthy and disease states and among different organs. Although phenotypic changes in ECs can promote healing in response to injury, excessive changes are also linked to various types of vascular pathology, including atherosclerosis, fibrosis after heart disease, and other vasculopathies.

**FIGURE 1:**
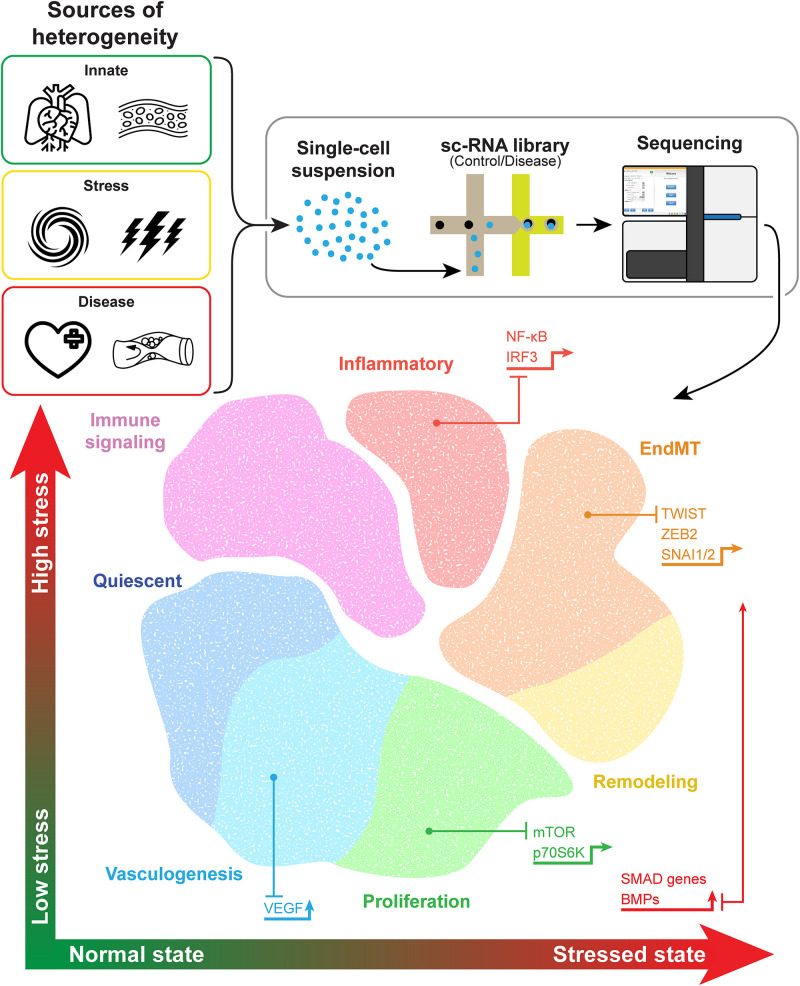
Endothelial cell heterogeneity revealed by cell-specific sequencing. Endothelial cells (ECs) exhibit phenotypic differences that stem from inherent organo-specific, vessel-specific, and site-specific functions. Additionally, ECs can change phenotypes in response to cell stress or vascular disease, with representative pathways and transcription factors promoting different phenotypes, as shown with the associated arrows. The isolation and sequencing of single cells allow for improved precision in determining different endothelial cell phenotypes or cell states. Although distinct phenotypes have been identified, some features overlap, such as proliferative stalk cells in vasculogenesis. Evidence supports a potential phenotypic spectrum of some types of ECs, such as those involved in remodeling and endothelial-mesenchymal transition.

Newer techniques such as single-cell analysis have provided even greater precision, improving our understanding of EC heterogeneity and how ECs change in response to stimuli. Data from single-cell RNA sequencing (scRNA-seq) analysis allow for unsupervised clustering of ECs on the basis of each cell’s transcriptome. Transcriptomic data collected from ECs in multiple different experiments and among several tissue types and disease states support the existence of a variety of EC phenotypes including quiescent ECs involved in maintaining homeostasis, proliferative ECs, inflammatory ECs, remodeling ECs, ECs involved in endothelial-mesenchymal transition (EndMT), and ECs involved in angiogenesis (Figure 1 and Table 1).

**TABLE 1:** Endothelial cell phenotypes identified by transcriptome analysis.

EC phenotype	Stimulus	Transcription factors	Genetic markers in scRNA-seq	EC function	References
Quiescent	Laminar flow	KLF2, KLF4	*Arhgap18, Adm, Apoc, Cd36, Gpihbp1, Hspb1, Lpl, Myip, Pltp, Scarb1*	Maintain homeostasis, lipid metabolism, vasculo-protection	Ohnesorge et al. (2010); Moonen et al. (2015); Schaum et al. (2018); Kalluri et al. (2019)
Proliferative	Disturbed flow, oxidative stress, vascular injury	YAP/TAZ	***Birc5***, *Cdca8, Ccna2, Ccdc34*, ***Cdc20***, *Cdkn1a, Cenpa*, ***Cks2***, *Cks1b, Ezh2, Fen1, H2afz, Hmgb2, Hmgn2, Hsp90aa1, Lgals1, Lig1, Lmnb1, Nrm, Pcna, Plvap*, ***Mki67***, *Rrm1, Rrm2, Smc2, Spc24, Stmn1, Tuba1b*,***Tyms***, ***Top2a***	Vessel repair after injury	Schaum et al. (2018); Khan et al. (2019); Kalucka et al. (2020)
Immune-signaling	Baseline immune surveillance, vascular injury		*Cxcl10*, ***Ifit1***,***Ifit2***, ***Ifit3***, ***Ifit3b***, *Igtp, Isg15, Stat1, Tgtp2*, ***Usp18***	Response to T-cells, response to viral proteins	Li et al. (2019); Kalucka et al. (2020)
Inflammatory	Disturbed flow, oxidative stress	IRF3, NF-kB	***Il1b***, *Il6, Ccl2, Ccl6, Ccl8, Cd74*, ***Cxcr4***, ***Icam1***, *Cd45*,***Tnf***	Immune cell recruitment	Vozzi et al. (2018); Tombor et al. (2021)
Remodeling	Disturbed flow		*Adamts genes, Anxa2*, ***Bgn***, ***Col5a***, *Emilin, Fnln2, Hmcn1, Mgp, Plat, Plau, Timp3*	ECM degradation and maintenance	Dolan et al. (2012); Kalluri et al. (2019); Li et al. (2019)
Endothelial-mesenchymal transition (EndMT)	Disturbed flow, vascular injury, TGF-β signaling	TWIST, SMAD3, SNAI1/2, ZEB2	***Col1A1***, ***Col1A2***, ***Col3A1***, *Ctgf, Fn1, Postn, Serpine1, Sele*	Vessel repair after injury, fibrosis	Kovacic et al. (2019); Xu et al. (2020); Tombor et al. (2021)
Vasculogenesis	VEGF, hypoxia, ischemia, disturbed flow		Tip cells:***Angpt2***, ***Apln***, *Adm, Cldn5*, ***Cxcr4***, ***Dll4***, *Ednrb, Efnb2, Esm1, Flt4, Gja4, Hey1, Igfbp3*, ***Jag***, *Kdr*, ***Kcne3***, *Nid2, Nrp1, Nrp2, Notch1*, ***Plxnd1***, *Ramp3*, ***Robo4***Stalk cells:*Ackr1, Ehd4, Ki67, Tmem176a/b, Tmem252, Selp, Vwf*	Create new capillaries, vascular healing, promote inflammation	Su et al. (2018); Zhao et al. (2018); Kalluri et al. (2019); Kalucka et al. (2020)

*Genes in bold are conserved in the same types of ECs across at least two studies. EC, endothelial cell; ECM, extracellular matrix.*

In this review, we summarize recent technological advancements that have improved our understanding of EC populations, the factors driving phenotypic change, and the ways in which ECs respond to their environment in health and disease.

## EC Phenotypes

### Quiescent ECs and the Promotion of Homeostasis

#### Cell-Specific Features

Quiescent ECs are common within the healthy vessel wall and promote homeostasis through metabolism, vasodilation, anticoagulation, and anti-inflammation (Ricard et al., 2021). In scRNA-seq studies, clusters associated with normal EC homeostasis have been identified that exhibit increased expression of genes involved in promoting EC stability (*Arhgap18*, *Adm*, and *Hspb1*), as well as lipid metabolism (*Cd36*, *Gpihbp1*, *Lpl*, *Myip*, *Pltp*, and *Scarb1*) (Hippenstiel et al., 2002; Kalluri et al., 2019; Lay et al., 2019). Using scRNA-seq analysis, Lukowski et al. (2019) showed that most ECs in normal mouse descending aorta are in a quiescent G1 phase, which is further supported by the results of cell cycle analysis showing predominantly G1 markers and few proliferative G2M/S phase markers. In a study by Li et al. (2019), the largest cluster of ECs was found to be associated with homeostasis and did not differ in proportion between healthy and diseased cardiac tissues. In another study, which provided a single-cell atlas of mouse ECs, ECs involved in metabolism were identified among different tissue types, indicative of an important and conserved function most likely related to homeostasis and disease resilience (Kalucka et al., 2020; Ricard et al., 2021).

#### Functions

In healthy tissues, quiescent ECs remain in cell-cycle arrest. However, in the setting of environmental stressors such as disturbed flow or ischemia, ECs may be stimulated to proliferate and promote angiogenesis (Zecchin et al., 2017). Therefore, quiescent ECs may be considered as a baseline from which ECs can undergo phenotypic changes in response to different stimuli.

#### Regulatory Genes and Pathways

Normal laminar flow has been shown to result in EC quiescence, featuring the downregulation of genes associated with proliferation, inflammation, and apoptosis (Dolan et al., 2012). Protective pathways activated by normal laminar flow include the mitogen-activated protein kinase kinase 5-extracellular signal-regulated kinase 5 (MEK5-ERK5) pathway, which was shown to decrease EndMT and atherogenesis (Moonen et al., 2015). Kruppel-like factor (KLF) 2 and KLF4, downstream effectors of the MEK5-ERK5 pathway, have also been shown to have vasculoprotective effects (Ohnesorge et al., 2010; Moonen et al., 2015). The presence of quiescent ECs and ECs that promote homeostasis in different environments highlights their important functions. ScRNA-seq analysis of ECs has revealed clusters with the activation of gene pathways involved in maintaining homeostasis that are conserved across different tissue types, including the Wnt and mitogen-activated protein kinase (MAPK) pathways (Paik et al., 2020).

### Proliferative ECs

#### Cell-Specific Features

Proliferative ECs have been identified by their increased viability in culture, as well as through transcriptome analyses showing their increased expression of cell-cycle progression genes (Dolan et al., 2012; Vozzi et al., 2018; Khan et al., 2019; Li et al., 2019; Kalucka et al., 2020). ScRNA-seq studies have also identified clusters of proliferative ECs. In mouse tumor cells, specific EC clusters expressed increased levels of proliferation markers including *Cks2*, *Tyms*, *Birc5*, *Cdc20*, *Ccna2*, *Cdca8*, *Mki67*, *Top2a*, and *Rrm2* (Khan et al., 2019; Li et al., 2019). Furthermore, increased expression of these proliferation markers within a distinct subcluster of ECs may be responsible for the overall increased expression of proliferation markers observed in the bulk RNA sequencing of tumor ECs. These findings highlight the impact of more precise sequencing methods in determining the mechanism underlying disease. In a single-cell atlas of ECs among different types of mouse tissues, proliferative ECs were associated with genes such as *Ccdc34*, *Cdkn1a*, *Cks1b*, *Cks2*, and *Tyms*. ScRNA-seq analysis of murine cardiac tissues revealed a cluster of cardiac ECs expressing similar proliferative genes, such as *Mki67*, *Top2a*, *Cks2*, *Birc5*, and *Cdc20*), as well as *Plvap* (Li et al., 2019).

#### Functions

In a normal state, ECs have low proliferative qualities; however, single-cell analysis of normal tissues has revealed the existence of clusters with gene expression that is consistent with that of proliferative ECs, most likely in areas exposed to disturbed flow or in areas undergoing vasculogenesis. ECs in areas of oscillatory shear stress and disturbed flow have been found to have increased mitotic activity (Chien, 2003). Proliferative ECs are also seen in stalk cells in vasculogenesis (Mack and Iruela-Arispe, 2018). Proliferative ECs are activated in response to vascular injury, hypoxia, and ischemia (Dolan et al., 2012; Vozzi et al., 2018; Li et al., 2019). After MI, an increase in proliferative ECs has been observed in murine hearts (Tombor et al., 2021). EC proliferation may represent a mechanism for maintaining the structural and functional integrity of the endothelium. Further studies may help determine whether these ECs are predominantly a response to vascular stress or a key factor in the promotion of vascular disease.

#### Regulatory Pathways

Most studies have evaluated proliferative ECs in the setting of disturbed flow. Disturbed flow has been shown to activate yes-associated protein 1 (YAP) and transcriptional coactivator with PDZ-binding motif (TAZ) and ERK1/2 and induce EC proliferation (Bao et al., 2001; Wang et al., 2016; Nakajima and Mochizuki, 2017). Additionally, upon exposure to disturbed flow, bone morphogenic proteins (BMPs) stimulate SMAD genes, which modulate cyclin-dependent kinases to induce cell cycle progression. Zhou et al. (2012) showed that disturbed flow alone can also activate SMAD1/5, which in turn activates the proliferative factors mTOR and p70S6K. This results in the upregulation of cyclin A, downregulation of p21 and p27 (two inhibitors of cell cycle progression), and increased EC proliferation.

### Inflammatory ECs

#### Cell-Specific Features

Inflammatory, or activated, ECs have been identified by their increased expression of genes involved in inflammatory cell recruitment including *Cxc4*, *Icam1*, *Cd45*, *Tnf*, *Il1b*, and *Ccl8* (Vozzi et al., 2018; Li et al., 2019; Tombor et al., 2021). In a study by Tombor et al. (2021), scRNA-seq analysis early after myocardial infarction (MI) revealed increased populations of inflammatory cardiac ECs (*Tnf*, *Il1b*, *Ccl2*, *Ccl6*, *Ccl8*, and *Cd74*).

#### Functions

Similar to proliferative ECs, inflammatory and immune-response ECs appear to be stimulated by vascular injury and may be involved in the first steps of vascular repair. However, these ECs promote immune cell recruitment and vascular inflammation, which can cause thrombosis, lipid accumulation, and pathologic remodeling, contributing to the initiation and progression of vascular diseases such as atherosclerosis.

#### Regulatory Pathways and Transcriptional Factors

Endothelial cells have been shown to be activated to a proinflammatory state by shear stress, oxidative stress, C-reactive protein (CRP), and proinflammatory cytokines (Szmitko et al., 2003). Activation of ECs can lead to a positive feedback cycle and promote further inflammation. Activated ECs release proinflammatory molecules, causing the surrounding cells in the vessel wall to produce cytokines, in turn resulting in increased EC activation. The stimulation of ECs with interleukin (IL)1-β and tumor necrosis factor (TNF)-α induces increased expression of monocyte adhesion molecules, as well as the release of proinflammatory cytokines such as IL-18 and monocyte chemoattractant protein (MCP)-1, which further attract inflammatory cells. Increased levels of CRP seen in states of inflammation inhibit the release of nitric oxide (NO) from ECs and increase the expression of cytokines, monocyte adhesion molecules, MCP-1, and nuclear factor-κB (NF-κB) (Verma et al., 2002; Szmitko et al., 2003).

Nuclear factor-κB is a transcription factor activated by oxidative stress and shear stress and is responsible for the regulation of many proinflammatory genes, including those encoding TNF-α, IL-1, IL-8, and monocyte adhesion molecules in ECs (Xiao et al., 2014). Additionally, activation of pro-inflammatory transcription factor interferon regulatory factor (IRF)-3 by reactive oxygen species (ROS) results in increased expression of ICAM-1 (Mao et al., 2017).

### Immune-Signaling ECs

#### Cell-Specific Features

Other EC phenotypes involved in immune-cell signaling identified by using single-cell analysis include ECs involved in natural killer cell signaling (*Klra3*, *Klra9*, and *Klra10*) and ECs involved in the interferon signaling response (*Ifit1*, *Ifit2*, *Ifit3*, *Ifit3b*, *Usp18*, and *Cxcl10*) (Li et al., 2019). A cluster of ECs involved in the interferon response was also identified in the single-cell atlas of mouse tissues, characterized by the increased expression of *Ifit1*, *Ifit3*, *Ifit3b*, *Igtp*, *Isg15*, *Stat1*, *Tgtp2*, and *Usp18* (Kalucka et al., 2020).

#### Functions

Immune-signaling ECs appear to be involved in innate immune surveillance, viral responses, and signaling to natural killer cells and T-cells. Like inflammatory ECs, immune-signaling ECs appear to be stimulated by vascular injury, but they are also likely important in baseline cell signaling in healthy tissues. Tissues with increased proportions of immune-surveillance ECs at baseline included those most involved in the immune response, such as liver, spleen, and lung (Kalucka et al., 2020). Interferon signaling through interferon gamma can also result in increased inflammation and decreased extracellular matrix (ECM) production in ECs (Sprague and Khalil, 2009). Li et al. (2019) reported that after MI, a cluster of ECs involved in interferon signaling was increased.

### EndMT and Mesenchymal-Like ECs

#### Cell-Specific Features

Endothelial cells have also been shown to undergo a phenotypic switch toward a mesenchymal phenotype and gain features of other cell types such as fibroblasts or smooth muscle cells (SMCs) (Zeisberg et al., 2007; Ubil et al., 2014; Moonen et al., 2015; Kostina et al., 2016; Kovacic et al., 2019; Xu et al., 2020). By becoming more mesenchymal-like, ECs gain the ability to invade and migrate through the ECM and produce collagen and contractile proteins (Kovacic et al., 2019). In a scRNA-seq study comparing vascular ECs in mice exposed to disturbed flow or stable flow, a small cluster of ECs exposed to chronic disturbed flow had increased expression of a gene associated with disturbed flow (*Thsp1*) and SMC, fibroblast, and immune cell genes (*Acta2*, *Tagln*, *Dcn1*, and *Cd74*), consistent with EndMT (Andueza et al., 2020). In another scRNA-seq analysis of cardiac ECs in mice, a cluster of ECs consistent with EndMT was identified by the increased expression of *Col3a1*, *Fn1*, *Serpine1*, and markers of EC proliferation. An EC cluster tagged by the marker *CDH5* in EC fate–mapped mice also expressed these markers during the initial days after cardiac injury, consistent with EndMT (Tombor et al., 2021). This cluster, which increased 1 week after MI, returned to baseline later in the recovery course, suggestive of a transient EndMT state in response to injury (Tombor et al., 2021). This transient state, in which few EC-traced cells lost their EC markers entirely to become traditional fibroblasts, may explain the differences observed in studies of EndMT levels after vascular injury.

#### Functions

The effect of EndMT appears to be time dependent, with transient EndMT occurring in the immediate period after injury. Furthermore, chronic disturbed flow is associated with promotion of EndMT in atheroprone regions of the vasculature. This suggests that EndMT may be beneficial for repair after vascular injury but may also perpetuate vascular disease by increasing neointimal hyperplasia and fibrosis in areas of chronic stress (Andueza et al., 2020; Tombor et al., 2021). Additionally, the anti-thrombotic qualities expressed in ECs at baseline are diminished in EndMT, increasing the risk of thrombosis after injury (Hong et al., 2019).

#### Regulatory Pathways

EndMT has been shown to occur during embryonic development after injury in response to transforming growth factor (TGF)-β, Notch signaling, and inflammatory molecules (Kostina et al., 2016). Canonical TGF-β signaling is considered the driving force of EndMT (van Meeteren and ten Dijke, 2012). *TGFB2* induces EndMT and the increased expression of SMC markers, which reverts to normal levels after *TGFB2* is withdrawn, supportive of a transient phenotype (Tombor et al., 2021). The binding of TGF-β to its receptor activates SMAD genes, promoting the activation of transcription factors associated with EndMT including *TWIST*, *SMAD3*, *ZEB2*, and *SNAI1/2* (Kovacic et al., 2019). Other regulatory pathways involved in EndMT including metabolic, non-coding RNA, and epigenetic regulation pathways are summarized elsewhere in a comprehensive review of EndMT by Kovacic et al. (2019).

### Remodeling ECs

#### Cell-Specific Features

Endothelial cells have been identified that have increased expression of ECM-remodeling genes, such as *ADAMTS* genes, *TIMP3*, and tissue plasminogen activators *PLAT* and *PLAU* (Dolan et al., 2012). In a scRNA-seq study of cardiac ECs, a cluster of ECs was identified with increased expression of ECM-associated genes, such as *Fbln2*, *Anxa2*, *Col5a2*, *Emilin*, *Hmcn1*, *Bgn*, and *Mgp* (Li et al., 2019). Another scRNA-seq analysis revealed a cluster of ECs with the increased expression of ECM genes, such as *Bgn*, *Dcn*, *Fn1*, *Fbln5*, *Lox*, *Mfap5*, *Pcolce2*, and several collagen genes.

#### Functions

Remodeling ECs are identified by genes associated with the degradation of ECM components (e.g., genes encoding fibrin, fibronectin, laminin, elastin, and collagen). These may be present in healthy tissues at baseline to assist in cell turnover and in response to laminar flow, whereby ECs migrate and realign to the direction of flow (Brooks et al., 2002; Conway et al., 2010). Remodeling ECs may also be involved in the early steps of angiogenesis, such as ECM breakdown and the rapid assembly/disassembly of focal adhesion molecules to assist with EC migration in vascular sprouts.

Additionally, remodeling ECs may be involved in repair after vascular injury without fully transitioning to an EndMT phenotype. In one study, scRNA analysis of ECs in a murine model of cardiac ischemia revealed a cluster of remodeling ECs that was increased after MI (Li et al., 2019).

Remodeling ECs appear to be involved in both normal vessel homeostasis and in response to cardiovascular injury. Because there is significant overlap in cluster-defining genes between ECs and EndMT cells, additional cell-specific transcriptome studies are needed to help determine whether this is truly a distinct EC phenotype or an early precursor to EndMT cells that respond to cardiovascular injury.

### ECs Involved in Angiogenesis

#### Cell-Specific Features

Phenotypic changes in ECs have been well studied in angiogenesis (Potente et al., 2011; Eelen et al., 2015; Mack and Iruela-Arispe, 2018; Mühleder et al., 2021). During capillary vessel sprouting, ECs can be divided into tip and stalk cells. Tip cells have transcriptome patterns of increased pro-angiogenesis factor expression and NOTCH signaling, whereas stalk cells are more highly proliferative (Mack and Iruela-Arispe, 2018). Single-cell analysis of ECs supports these phenotypes. A study of tumor ECs revealed a cluster with elevated expression of tip cell markers *Kcne3*, *Nid2*, and *Dll4*, as well as a cluster with increased expression of stalk cell markers *Vwf* and *Selp* (Zhao et al., 2018). Intermediate cells that fall between a tip and stalk cell phenotype were also identified. Several markers overlapped with the clusters identified by Li et al. in their single-cell analysis of cardiac tissue, which included one cluster consistent with stalk cells (*Ackr1*, *Ehd4*, *Tmem176a/b*, *Tmem252*, and *Selp*) and one cluster consistent with tip cells/NOTCH signaling (*Dll4*, *Notch1*, *Hey1*, *Jag1*, and *Gja4*) (Li et al., 2019). In another single-cell study of ECs, two EC clusters were identified that were involved in angiogenesis: one expressed genes consistent with tip cells (*Cxcr4*, *Cd34*, *Dll4*, *Flt4*, *Nrp1*, *Nrp2*, and *Plxnd1*), and the other expressed genes consistent with stalk cells (Kalluri et al., 2019).

#### Functions

In healthy vascular tissue, angiogenesis is important for blood vessel repair and new vessel formation. However, in vascular disease, angiogenesis can occur in response to environmental stimuli such as vessel injury or hypoxia. Pathologic increases in angiogenesis are also seen in tumor vasculature. Angiogenesis and inflammation may be linked, resulting in a positive feedback loop and the progression of vascular disease (Jeong et al., 2021).

#### Regulatory Pathways

Angiogenesis is stimulated by vascular endothelial growth factor (VEGF). ECs with the highest expression of the receptor gene *VEGFR2* have the greatest pro-angiogenesis response and lead to new vessel formation as tip cells (Eelen et al., 2015). Increased NOTCH signaling from tip cells via *Dll4* results in decreased ERK activity in stalk cells, causing decreased cell-cycle inhibition and increased proliferation (Mühleder et al., 2021). Anti-angiogenesis treatments that block *VEGF* and *Dll4* reduce the proliferation of ECs involved in capillary vessel sprouting, particularly tip cells (Zhao et al., 2018).

## Innate Contributions to EC Heterogeneity

Environmental stimuli have an important effect on the regulation of EC phenotypes (Aird, 2012). In addition, EC phenotypes differ widely, including organo-specific ECs, location-specific-ECs, and disease-specific ECs. Although these phenotypes may overlap, it is important to understand phenotypic changes within the context of the tissue type and conditions of each experiment. ECs from different types of vessels and tissues have been shown to have unique transcriptomes (Zhao et al., 2018; Kalucka et al., 2020; Paik et al., 2020). Different artery locations expose ECs to different stimuli, such as increased oxygen levels or mechanical stress. Cultured ECs have been shown to have expression patterns that are different than those observed *in vivo.* For example, differences in gene expression between ECs of different arteries *in vivo* are not as evident in cultured ECs (Burridge and Friedman, 2010). Furthermore, differential gene expression between lymphatic ECs and blood vessel ECs is also diminished (Wick et al., 2007). This indicates the importance of environment on EC gene expression. However, cells do retain some distinguishing factors *in vitro*, such as intrinsic transcriptional profiles stemming from vascular bed origin and vessel location, which is supportive of a multifactorial process that leads to cell-specific EC transcriptomes (Wick et al., 2007; Burridge and Friedman, 2010; Kutikhin et al., 2020).

### Phenotypic Spectrum of ECs Across Different Vascular Beds

Endothelial cells exhibit unique expression profiles associated with vessel type in four main vessels: arteries, veins, capillaries, and lymphatic vessels. Cell studies conducted *in vitro* have identified EC precursors that express markers of different vessel beds. For example, *Hey2*, *Nos3*, and *Flt1* are expressed as arterial markers; *Klf4* and *Cdh2* as venous markers; and *Lyve1* as a lymphatic EC marker (Kutikhin et al., 2020). In a single-cell analysis of mouse embryonic tissues, arterial and venous populations of ECs were identified that had distinct gene expression profiles. Using single-cell analysis, Kalucka et al. (2020) showed that gene expression across ECs in arteries, capillaries, and veins occurs along a phenotypic spectrum. The highest expression of the traditional EC markers *Vwf* and *Vcam1* was seen at either end of the spectrum, consistent with large vessel arteries or veins. In this study, the EC transcriptomic signatures that were most consistent with large arteries across different tissue types (liver, lung, heart, and kidney) included *Gja4*, *Fbln5*, and *Clu*, whereas those most consistent with large veins included *Apoe*, *Bgn*, and *Plvap*. Some markers such as *Mgp*, which is an inhibitor of tissue calcification, were found to be specific to both arteries and veins depending upon tissue type. Another analysis, in which endothelial-specific translating ribosome affinity purification (EC-TRAP) and high-throughput RNA sequencing were used, *Ephb4* was identified as a marker for arteries, and *Dll4* as a marker for capillaries (Cleuren et al., 2019).

Of the vessel-specific ECs, heterogeneity among tissue types was highest among capillary ECs, which had the fewest conserved markers across different tissue types, indicating that phenotypic changes in capillary ECs may be responsible for most of the adaptive response to tissue- and environment-specific stimuli. The ability of ECs to change from venous to arterial ECs has also been observed in single-cell studies of EC development (Su et al., 2018).

Lymphatic ECs have been previously identified by the increased expression of *Lyve1* and *Pdpn* and appear distinct from blood vessel ECs (e.g., artery, vein, and capillary ECs) on single-cell analysis (Wick et al., 2007; Kalucka et al., 2020). When multiple datasets from the single-cell analysis of ECs were combined, lymphatic ECs remained clustered together across different tissue types and experiments, indicating a highly conserved and specific transcriptome (Feng et al., 2019). In a scRNA-seq study of lung, heart, liver, and intestinal tissue, a cluster of lymphatic ECs was identified that expressed conserved genes including *Ccl21a*, *Mmrn1*, *Fgl2*, and *Thy1* (Kalucka et al., 2020). In another scRNA-seq study of mouse aorta, a cluster of lymphatic ECs was identified that expressed *Lyve1*, *Prss23*, and *Fxyd6* (Kalluri et al., 2019).

### EC Atlases of Multiple Organ Types

As more single-cell studies of ECs are completed, publicly available data can be combined to support more powerful analyses with greater cell counts. A single-cell atlas of murine EC transcriptomes has been formed, and data have been combined for multiple different tissue types. These studies have provided insight into tissue- and vessel-specific transcriptomes, as well as sex-based differences in ECs, supporting the existence of extensive EC heterogeneity (Schaum et al., 2018; Kalucka et al., 2020; Paik et al., 2020). Transcriptional profiles for ECs in different tissues are unique, indicating tissue-specific EC functions, such as roles in membrane transport across the blood-tissue barrier in brain and testes, immunoregulation in spleen and liver, and redox homeostasis in heart tissues (Kalucka et al., 2020; Paik et al., 2020).

When comparing cardiac to vascular ECs, scRNA-seq revealed valvular, coronary artery, and heart wall ECs as clusters separate from vascular ECs (Cui et al., 2019; Feng et al., 2019). *NPR3* was found to be specific to cardiac ECs, whereas vascular ECs were found to have increased expression of ECM genes including *ELN*, *FBLN2*, and *FBLN5* (Cui et al., 2019). *Edh3* and *Fam167b* were identified as aorta-specific EC markers (Feng et al., 2019).

However, some transcriptional profiles are similar among tissues including adipose, heart, aorta, and kidney, indicating that there is at least some degree of overlap and that transcriptional differences seen within one organ system should be considered as potential differences in other systems. Additionally, the overlap of transcriptional phenotypes observed between mouse and human ECs within the same tissue type supports the translation of findings from mouse models to human disease.

### Contribution of Sex-Based Differences to EC Heterogeneity

Sexual dimorphism in ECs has been observed and provides insight into sex-based differences that may affect the risk of vascular diseases (Stanhewicz et al., 2018). Compared with men, premenopausal women with hypertriglyceridemia were found to have increased levels of progenitor ECs, along with enhanced production of nitric oxide required for vascular homeostasis, indicating a protective role for these ECs against vascular disease in women (Ren et al., 2020). Additionally, female cultured human umbilical ECs showed higher expression of genes involved in cell proliferation and migration than did male human umbilical ECs. Thus, even *in vitro*, attention should be given to whether cells originate from males or females, and cells from both sexes should ideally be used for *in vitro* studies (Addis et al., 2014). Paik et al. (2020) performed scRNA-seq analysis of mouse ECs from different tissue types and also identified cluster-specific sex-based differences in ECs among mouse tissues, most predominantly in adipose, heart, and kidney tissues. This further emphasizes the importance of studying ECs from both sexes, particularly at a single-cell level, and supports the notion of different transcriptional patterns between sexes as a driving factor in vascular disease.

## Effects of Biomechanical and Biochemical Stress on ECs

### The Dose-Dependent Response of ECs to Laminar Shear Stress

Normal laminar flow has been shown to protect against vascular disease, whereby normal laminar flow stimulates EC quiescence and inhibits factors that promote inflammation, such as the Hippo/YAP pathway (Yuan et al., 2020). Dose-dependent gene expression within ECs at different levels of shear stress has been reported and is associated with vascular protection against higher shear stress (Brooks et al., 2002; Ohura et al., 2003; Conway et al., 2010; Dolan et al., 2012; Vozzi et al., 2018). The expression of flow-dependent genes *KLF2* and *KLF4* was not found to increase with increasing laminar flow, but the expression of other genes including inhibitory SMAD genes *SMAD*6 and *SMAD7* and the nitric oxide synthase gene *NOS3* increased with the level of flow (Mandrycky et al., 2020). In a scRNA-seq analysis of cultured ECs, cells exposed to high laminar flow became quiescent and more homogenous in phenotype (Helle et al., 2020). Compared with static conditions, low levels of shear stress were shown to increase EC viability and the expression of EC genes involved in mitosis, indicating a proliferative response (Brooks et al., 2002; Conway et al., 2010). However, as shear stress was increased, EC viability and markers of proliferation decreased, indicating that ECs exhibit a dose-dependent response to shear stress in proliferative pathways (Ohura et al., 2003; Vozzi et al., 2018; Helle et al., 2020). In the single-cell analysis of ECs exposed to high flow, a spectrum of ECs was identified, with highly proliferative cells on one end and quiescent cells on the other. Many genes in the quiescent cluster were those regulated by Notch pathway activation, such as that seen in laminar shear stress (Mandrycky et al., 2020). Furthermore, under high shear stress conditions, the expression of ECM remodeling genes *MMP2* and *MMP9* was also increased (Sho et al., 2002; Dolan et al., 2012; Mahmoud et al., 2017), and the expression of genes associated with cell-matrix adhesion was decreased (Dolan et al., 2012). This supports previously reported findings that high shear stress induces EC rearrangement, such as ECs becoming elongated and aligning with the direction of flow (Brooks et al., 2002; Conway et al., 2010).

### The EC Response to Disturbed Flow

Although changes in EC gene expression have been reported for static-, low-, and high-flow states, disturbed flow is most commonly associated with vascular diseases, such as atherosclerosis (Brown et al., 2016). Shear stress due to disturbed blood flow has been implicated as a driving factor for phenotypic changes in ECs, as evidenced by the unique transcriptional profiles of cells subjected to disturbed flow compared with those subjected to both high and low laminar flow states (Brooks et al., 2002; Conway et al., 2010; Vozzi et al., 2018; Andueza et al., 2020). EC proliferation is triggered by disturbed flow, as shown by the high proportion of ECs in mitosis in areas of disturbed blood flow (Zhou et al., 2012; Lee and Chiu, 2019). Additionally, disturbed flow but not laminar flow has been shown to lead to EndMT, resulting in neointimal hyperplasia and atherogenesis (Moonen et al., 2015). In a study by Andueza et al. (2020), scRNA-seq revealed clusters in ECs exposed to acute disturbed flow that were distinct from clusters in ECs exposed to chronic disturbed flow, indicating a time-dependent relationship between ECs and disturbed flow, with chronic disturbed flow being more closely associated with EndMT. Interestingly, in this analysis, some clusters of ECs were identified as more responsive than others to changes in flow states, suggesting that different EC phenotypes may have varying responses to shear stress and that a specific group of ECs may drive inflammatory and pathologic changes (Andueza et al., 2020). ScRNA-seq analysis showed the upregulation of inflammatory markers in ECs exposed to disturbed flow, particularly chronic disturbed flow (Andueza et al., 2020). Inflammatory adhesion was also highest in flow reversal states that mimic arterial disturbed flow *in vitro* (Conway et al., 2010; Vozzi et al., 2018).

Single-cell analysis of ECs has revealed the presence of quiescent, proliferative, inflammatory, and remodeling EC populations in disease states and under normal conditions. These populations may represent different stages of the endothelial response to laminar and disturbed flow. For example, laminar flow may promote a quiescence phenotype as well as a remodeling phenotype in ECs to allow for realignment with the direction of flow, whereas disturbed flow may promote proliferative, inflammatory, and EndMT phenotypes in ECs. These populations may also represent ECs in different physical locations, with some ECs having greater exposure to changes in flow, or some ECs being predisposed to respond to flow. Future studies with a focus on identifying where each of these unique cell populations are located may reveal cell populations or locations that are at increased risk for a pathologic response to shear stress. Precision spatial genomics provides a method to identify these populations *in situ* (Eng et al., 2019).

### EC Changes in Response to Oxidative Stress

Endothelial cells also react to other environmental stressors, such as oxidative stress. Oxidative stress may be associated with risk factors for vascular disease such as smoking, hypertension, and hyperlipidemia. In future cell-specific studies, analyzing the changes in ECs of patients with risk factors for vascular disease may provide more information about how ECs respond to oxidative stress. In previous studies, ROS have been linked to inflammatory gene expression and the promotion of monocyte infiltration into the vascular wall (Kunsch and Medford, 1999). Oxidative stress may upregulate EC expression of the CD40 receptor, a member of the TNF family, and result in CD40-mediated EC activation, which stimulates ROS production, increased leukocyte adhesion, and inflammatory cell recruitment (Nakanishi et al., 2001; Schönbeck and Libby, 2001; Urbich et al., 2002).

## EC Phenotypes in Cardiovascular Disease

Endothelial cell clusters unique to vascular disease have been identified. To date, most EC-specific studies have been performed in experimental models of atherosclerosis and heart disease (Li et al., 2019; Andueza et al., 2020; Tombor et al., 2021).

### Atherosclerosis

Areas of vessels with disturbed flow are more likely to develop atherosclerosis, such as the carotid bifurcations, aortic arch, and peripheral arterial branch points (Lee and Chiu, 2019). Analysis of EC phenotypes seen in vessels with disturbed flow support the observed increase in proliferative, inflammatory, and EndMT ECs in response to disturbed flow compared with laminar flow (Zhou et al., 2012; Moonen et al., 2015; Andueza et al., 2020). Cell-specific analysis of ECs in human atherosclerotic plaque has shown a transition to inflammatory and EndMT phenotypes (Depuydt et al., 2020).

In an scRNA-seq study (Andueza et al., 2020), EC phenotypes associated with acute disturbed flow were marked by expression of *Ctfg*, *Serpine1*, and *Edn1*. EC phenotypes associated with chronic disturbed flow were marked by *Thsp1*. A cluster of ECs involved in EndMT after exposure to chronic disturbed flow was marked by *Acta2*, *Tagln*, *Dcn1*, and immune cell markers including *CD74*. Transcription factor motifs associated with disturbed flow included *KLF4*, *RELA*, *AP1*, and *STAT1*. Additionally, in a scRNA-seq study (Depuydt et al., 2020) of atherosclerotic plaque, activated ECs were marked by *CD34*, *PECAM1*, *TIE1*, *ACKR1*, *PRCP*, and *VCAM1*. EndMT ECs in atherosclerotic plaque were marked by *ACTA2*, *NOTCH3*, and *MYH11*.

### Cardiac Disease

Single-cell analysis of murine cardiac ECs after MI revealed an increased proportion of ECs involved in the inflammatory response, proliferation, interferon signaling, vasculogenesis, and remodeling after MI compared with controls. Tombor et al. (2021) reported an increase in EC phenotypes involved in inflammation and proliferation as well as an increase in EndMT in murine cardiac ECs after MI. These EndMT clusters returned to baseline later in the recovery course, suggestive of a transient EndMT state in response to injury (Tombor et al., 2021). However, not all scRNA-seq analyses of ECs in mice after MI show an increase in EndMT during repair after injury (Li et al., 2019), most likely because of variations in the degree of EndMT transition (Kovacic et al., 2019; Tombor et al., 2021). This transient state identified by Tombor et al. (2021), in which very few EC-traced cells lose their EC markers entirely to become traditional fibroblasts, may explain differences between studies of EndMT levels after vascular injuries.

Single-cell analysis of murine hearts after MI revealed the upregulation of *Col3A1*, *Fn1*, and *Serpine1* in EC clusters undergoing transient EndMT and the downregulation of EC marker genes such as *Cdh5* and *Cd36* (Tombor et al., 2021). Overall, the gene ontology terms upregulated in these clusters included ECM organization, collagen synthesis, metabolic activity, angiogenesis, and Wnt signaling. EndMT transition in MI has been associated with Wnt signaling, with ECs that have undergone EndMT marked by increased expression of contractile genes and genes associated with Wnt signaling for 4 days after MI (Aisagbonhi et al., 2011; Tombor et al., 2021).

### Neointima Formation and Restenosis

Endothelial cell dysfunction and remodeling are associated with neointima hyperplasia after injury or with restenosis after vascular procedures (Douglas et al., 2013; Moonen et al., 2015). Although neointimal hyperplasia has largely been considered to be due to SMC proliferation, increased attention has been placed on EC involvement. EC recruitment of inflammatory cells as well as EC proliferation and EndMT may contribute to the promotion of neointimal formation (Moonen et al., 2015; Moser et al., 2016).

On histologic analysis, ECs have been found to have an invasive phenotype in neointima formation, with most ECs remaining as ECs rather than undergoing EndMT (Yuan et al., 2017). Performing single-cell analysis of vascular tissues after procedures (e.g., cardiac stents or endovascular procedures) may help identify more precisely the EC-specific changes involved in restenosis and whether there are small populations with pro-inflammatory, proliferative or EndMT phenotypes, as seen in atherosclerotic plaque or after MI.

### Calcific Aortic Valve Disease

Endothelial cells have been shown to have an important role in calcific aortic valve disease. As observed in atherosclerosis, ECs exposed to mechanical or shear stress become dysfunctional, with increased lipid deposition and immune cell infiltration (Goody et al., 2020). Calcification of the valve is more commonly found on the fibrosa (outflow) side of the valve, in the area most exposed to disturbed flow. Concordantly, EndMT transition of valvular ECs (VECs) has been identified as a factor in the progression of disease (Ma et al., 2020).

Early changes in human calcific aortic valve ECs consistent with EndMT have been identified by using single-cell analysis (Xu et al., 2020). In this study, one of the clusters of VECs was found to be an early cluster along a trajectory leading to traditional vascular interstitial cells, indicative of EndMT transition in calcific aortic valve disease.

Single-cell analysis of calcific aortic valve disease revealed that two clusters of VECs expressing the marker genes *SELE*, *SERPINE1*, *IL1R1*, and *PI3* were involved in EndMT (Xu et al., 2020). These clusters showed enrichment of the PI3K-Akt and Wnt pathways, which have been found to have a role in EndMT, whereby their upregulation promotes mesenchymal transition (Meadows et al., 2009; Zhong et al., 2018). Additionally, an *in vitro* study of aortic VECs showed that UBE2C (ubiquitin E2 ligase C) responded to disturbed flow by increasing *HIF1*α expression to promote VEC EndMT (Fernandez Esmerats et al., 2019).

### Aortic Aneurysms

Much of aortic aneurysm research is focused on SMC mutations and ECM changes. However, because ECs are the first cells to be exposed to environmental stressors and to induce the activation of signaling pathways within the aortic wall, EC phenotype change in aortic aneurysms is an important area of study. Bicuspid aortic valves have been associated with ascending thoracic aortic aneurysms (ATAAs), and the disturbed flow through the valve may stimulate ECs to become proliferative and pro-inflammatory and to undergo EndMT, leading to vascular remodeling and aortic aneurysm formation (van de Pol et al., 2017). ECs are also involved in the angiotensin II signaling pathway, which has been linked to aneurysm development. The endothelial-specific knockout of the angiotensin II *AT1* receptor was shown to protect against thoracic aortic aneurysms (Rateri et al., 2011).

#### Genes Associated With Aortic Aneurysm Formation in ECs

Few single-cell sequencing studies have focused on EC phenotypes in aortic aneurysm development. In a study of sporadic ATAA by Li et al. (2020), two clusters of ECs were identified. Upon the analysis of differential gene expression, one of the clusters was found to have the majority of differentially expressed genes between sporadic ATAA and control tissues. In this cluster, the transcription factor *ERG* was found to be downregulated in ATAA tissues, along with its downstream genes involved in apoptosis and the response to reactive oxygen species, suggesting that *ERG* may be pathologically downregulated in ATAA.

## Conclusion

Endothelial cells have dynamic populations with diverse functions. While these functions may overlap, ECs do have distinct transcriptomes, as highlighted by scRNA sequencing. Changes in EC phenotype are influenced by microenvironment, innate organo- and lineage-specific factors, and biomechanical and biochemical stressors.

Alteration of EC gene expression is an early event in vascular disease and plays a critical role in its progression. Identification of potential pathologic phenotypes may help advance precision medicine and guide cell-specific therapies in vascular disease. EC phenotypes identified on scRNA sequencing that are predominantly associated with cell stress include proliferative, inflammatory, and EndMT phenotypes, which may be pathologic if overexpressed. Additionally, knowledge of organo- and lineage-specific transcriptomic signatures may ultimately improve bioengineered tissues, including vascular conduits, and the organo-specific targeting of treatments.

Future studies will be important for expanding upon these phenotypes in different conditions. As more data become available, combining publicly available data obtained under similar conditions will increase the power and precision of analyses.

## Author Contributions

All authors conceived and designed the review, wrote the manuscript, participated in revising the manuscript, and read and approved the final version of the manuscript.

## Conflict of Interest

The authors declare that the research was conducted in the absence of any commercial or financial relationships that could be construed as a potential conflict of interest.

## Publisher’s Note

All claims expressed in this article are solely those of the authors and do not necessarily represent those of their affiliated organizations, or those of the publisher, the editors and the reviewers. Any product that may be evaluated in this article, or claim that may be made by its manufacturer, is not guaranteed or endorsed by the publisher.
